# Bacterioplankton Responses to Increased Organic Carbon and Nutrient Loading in a Boreal Estuary—Separate and Interactive Effects on Growth and Respiration

**DOI:** 10.1007/s00248-017-1115-7

**Published:** 2017-12-18

**Authors:** Ana R. A. Soares, Emma S. Kritzberg, Ioana Custelcean, Martin Berggren

**Affiliations:** 1 Department of Physical Geography and Ecosystem Science, Lund University, SE-223 62 Lund, Sweden; 2 Department of Biology/Aquatic Ecology, Lund University, SE-223 62 Lund, Sweden

**Keywords:** Bacterial production, Terrestrial dissolved organic matter, Nutrient limitation, Nutrient interactions, Bacterial respiration, Bacterial metabolism

## Abstract

Increases in the terrestrial export of dissolved organic carbon (C) to rivers may be associated with additional loading of organic nitrogen (N) and phosphorus (P) to the coastal zone. However, little is known about how these resources interact in the regulation of heterotrophic bacterioplankton metabolism in boreal coastal ecosystems. Here, we measured changes in bacterioplankton production (BP) and respiration (BR) in response to full-factorial (C, N, and P) enrichment experiments at two sites within the Öre estuary, northern Sweden. The BR was stimulated by single C additions and further enhanced by combined additions of C and other nutrients. Single addition of N or P had no effect on BR rates. In contrast, BP was primarily limited by P at the site close to the river mouth and did not respond to C or N additions. However, at the site further away from the near the river mouth, BP was slightly stimulated by single additions of C. Possibly, the natural inflow of riverine bioavailable dissolved organic carbon induced local P limitation of BP near the river mouth, which was then exhausted and resulted in C-limited BP further away from the river mouth. We observed positive interactions between all elements on all responses except for BP at the site close to the river mouth, where P showed an independent effect. In light of predicted increases in terrestrial P and C deliveries, we expect future increases in BP and increases of BR of terrestrially delivered C substrates at the Öre estuary and similar areas.

## Introduction

Despite representing a small area of the global ocean, coastal zones are major hotspots for biogeochemical cycling of the macroelements such as organic carbon, nitrogen (N), and phosphorus (P) in marine environments. The availability of N and P is central to both algal primary production and bacterioplankton secondary production (BP), and thus, these nutrients support the basis of marine food webs, exerting bottom-up control of the structure and function of coastal ecosystems [1, 2]. In addition, the input of dissolved organic carbon (C) fuels bacterial respiration (BR), contributing to the production of dissolved carbon dioxide and to the consumption of oxygen [3]. Because the input fluxes of C, N, and P control much of the structure and functioning of coastal marine systems, it is of major importance to predict the changes in these fluxes, e.g., due to climate or land use change [4, 5].

Changes in climate are predicted to result in a 30% increase in precipitation in the northern Baltic Sea region during the current century [6]. Subsequently, a larger amount of terrestrial dissolved organic carbon (DOC) will reach the coastal zone due to increased terrestrial runoff [7]. In addition, increased nutrient loading may occur [5, 8–12]. Such nutrients include N and P bound to the humic fraction of the terrestrially derived DOC, which may become available to estuarine microbes through enzymatic or photochemical processing [13, 14]. In boreal unproductive estuaries where heterotrophic bacterioplankton rely on the supply of terrestrial substrates, such as the Öre estuary in the northern Baltic Sea [15], increased export of DOC and nutrients is expected to have profound consequences on bacterioplankton metabolism, i.e., BR and BP [16–18].

Bacterioplankton metabolism is often limited by C, N, and P in aquatic systems [3]. To support BR, C availability is critical as bacteria derive energy from organic substrates. Organic carbon can also be used to support BR even during periods of nutrient limitation of growth [3]. Particularly in the Baltic Sea, empirical evidence has shown increased BR in response to additions of riverine dissolved organic matter [DOM; 16]. Organic carbon is also a well-known regulator of BP in many coastal seas [19, 20] and C limitation of BP has been previously reported in the Baltic Sea [18, 21, 22]. However, BP is often primarily limited by N and P due to the large demand for these elements to sustain high protein, phospholipid, and nucleic acid production in growing bacterial cells [3, 23]. Bacterial production in the Baltic Sea has been shown P-limited [24] or co-limited by C and nutrients [25], with varying limitation patterns in time and space [26]. Typically, the high freshwater inflows in the northern Baltic Sea, characterized by high TN/TP ratios, drive BP towards P limitation in coastal areas [24, 27]. Offshore and southern parts of the Baltic Sea have been shown N limited [28, 29]. Nutrient limitation commonly occurs during the productive season, while C limitation of BP takes place during pre- and post-bloom periods [25]. Due to low rates of primary productivity at the Öre estuary, the import of terrestrial C is essential to estuarine bacteria particularly in periods other than the productive season [15]. Bacterial limitation thus varies seasonally, following periods of either nutrient or C limitation depending on in situ primary production and on external delivery of C and nutrients.

Despite expected increases in C, N, and P loading, few studies to date have tested how estuarine bacterial metabolism will respond, and how this response will vary temporally and spatially. Existing knowledge on C, N, and P controls on estuarine bacterioplankton metabolism is mostly based on correlational patterns, which do not express causal relationships nor identify the interactive effects of the different elements. Because coasts are highly heterogenic systems, there is need for a mechanistic understanding of resource control on bacterioplankton metabolism, allowing the extrapolation of coastal element cycling dynamics from small to regional scales. Enrichment bioassays can be applied to investigate patterns of resource limitation, yet few have been conducted in boreal estuaries. This limited knowledge precludes an adequate understanding of the C, N, and P cycling at the estuarine interface between land, ocean, and atmosphere compartments.

In this study, we determined potential resource limitation patterns. We aimed to determine single and interactive short-term effects of increasing C, N, and P on estuarine BR and BP during one complete year. The study was performed in the Öre estuary (northern Baltic Sea), which is highly influenced by riverine DOM loadings, which are projected to increase further [30]. Because increases in BR are mainly dependent on C availability, we hypothesized that single additions of C will increase BR independently of the increase in nutrients. Secondly, because BP is theoretically dependent on both C and nutrients (especially P) availability, we hypothesized that BP will increase only if C and P are added in combination. Thirdly, because C, N and P often limit bacterial metabolism across aquatic systems, we hypothesized that the combined increase of C, N, and P will have a three-way positive interaction effect on both BR and BP. To test our hypotheses, we employed in vitro full-factorial amendments of C, N, and P and assessed their effect on BP and BR during 72 h. Additionally, we performed an explorative analysis of how changes in the environmental conditions affect element limitation patters in the estuary over space and time.

## Methods

### Study Area and Sampling

**Fig. 1:**
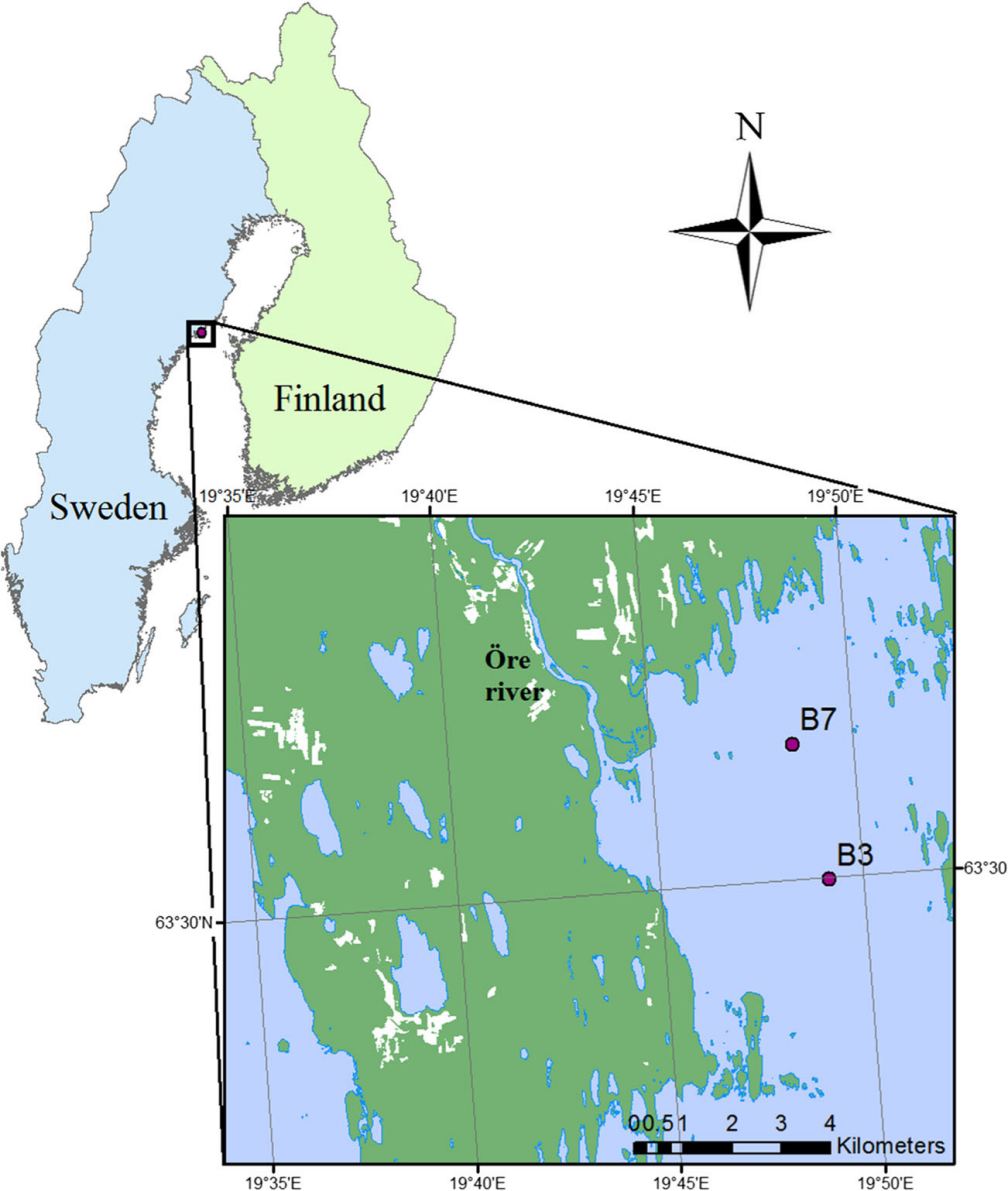
Map of the Öre Estuary including the two sampled sites B7 (63° 31.50 N, 19° 48.49 E) and B3 (63° 29.98 N, 19° 49.14 E)

The study took place at the Öre estuary, located in the northwestern part of the Bothnian Sea, which is one of the sub-basins of the Baltic Sea (Fig. 1). Terrestrially derived DOM transported through river runoff represents the highest organic matter input in the estuary, particularly during high flow periods such as snowmelt (which usually occurs around May) and during autumn [31]. The estuary represents a semi-enclosed area with no tides, where effects of terrestrially derived DOC are strong [9]. The estuary has an approximate area of 50 km^2^, mean depth of 16 m and a water volume of 1.0 × 10^9^ m^3^ [32]. Water residence time within the estuary is variable, but the near-sea mixed layer of the estuary can overturn in less than 2 weeks due to exchange of water with the Bothnian Sea basin [33]. The salinity ranges from 1 at the inner part of the estuary to 5 at the outer part.

Freshwater inputs from the Öre catchment are unregulated, and average discharge is approximately 365 mm per year. The Öre catchment has a total area of 3002 km^2^. The catchment is dominated by a large fraction of coniferous forests (approximately 83%), mainly Norwegian spruce (*Picea abies* L. Karst.) and Scots pine (*Pinus sylvestris* L.), and by a substantial fraction of mires (12%; estimates provided by the Swedish Meteorological and Hydrological Institute, SMHI). The catchment has low influence of industrial sources, agricultural areas, and wastewater treatment plants along the river [32]. Total river length is 225 km [33].

Two estuarine sites, which are part of the Swedish national water quality monitoring program, were sampled by the Umeå Marine Sciences Centre (UMF) at 1m depth seven times each during a year (from March 2014 until March 2015). Both sampling sites are located in the central part of the estuary, and the distance between the two sites is approximately 3 km. One site is closer to the Öre river mouth (site “B7”; 63° 31.50 N, 19° 48.49 E; Fig. 1) than the other site (“B3”; 63° 29.98 N, 19° 49.14 E; Fig. 1), and on average, site B7 has lower salinity (3.5) than site B3 (4.1; Table 1). Water samples were kept cool during transport and stored refrigerated until the experiments started which occurred within 3 to 4 days after sampling.

**Table 1:** Estuarine physicochemical and biological variables at costal stations B7 and B3 during the experimental period. Chl-a, chlorophyll-a; DIN, dissolved inorganic nitrogen; PO_4_-P, phosphate; DON, dissolved organic nitrogen; DOP, dissolved organic phosphorus; TN, total nitrogen; TP, total phosphorus

Site and sampling number	Sampling date	River discharge (m^3^ s^−1^)	Salinity	Temperature(°C)	In situ BP (μg C L^−1^ day^−1^)	Chl-a (μg L^−1^)	DIN (μM)	PO_4_-P (μM)	DOC (μM)	DON (μM)	DOP (μM)	DOC:TN (M)	DOC:TP (M)	TN:TP (M)
B3_1	24/03/2014	20.4	4.6	0.23	1.57	1.52	5.52	0.01	358	9.90	15.41	23	1085	47
B7_1	09/04/2014	15.3	4.3	1.20	5.35	9.69	1.48	0.05		14.51	15.94			44
B7_2	05/05/2014	75.6	0.2	6.15	8.71	4.25	0.24	0.03	389	17.89	18.1	21	794	37
B3_2	19/05/2014	63.0	4.0	6.96	8.07	4.38	0.18	0.03	391	24.24	24.39	16	465	29
B3_3	12/06/2014	49.1	3.2	14.10	7.69	3.12	0.47	0.02	458	16.66	17.11	27	1579	59
B7_3	30/06/2014	16.7	3.4	12.00	11.33	2.01	0.17	0.01	370	15.54	15.7	24	1423	60
B7_4	14/08/2014	07.2	3.0	19.70	15.51	3.68	0.27	0.03	393	16.36	16.60	24	983	42
B3_4	27/08/2014	50.6	3.6	15.30	9.36	3.54	1.18	0.02		13.81	14.97			52
B7_5	09/10/2014	13.8	4.7	8.61	2.41	2.48	3.78	0.35	349	13.07	16.50	21	623	30
B3_5	20/10/2014	26.8	4.2	7.44	1.22	2.82	3.40	0.17		14.73	17.96			48
B7_6	01/12/2014	14.5	3.9	4.24	0.69	1.27	4.98	0.23	374	14.08	18.83	20	831	42
B7_7	12/01/2015	8.40	5.1	0.69	0.31	0.64	5.10	0.45	343	13.75	18.4	17	512	28
B3_6	09/02/2015	8.60	5.1	0.72	0.88	0.43	5.27	0.38	348	13.86	18.75	18	669	37
B3_7	09/03/2015	8.30	4.0	0.82	0.85	1.25	7.90	0.42		13.80	21.28			35

### Experimental Setup

After arrival at the laboratory, the samples were filtered on a 1.2-μm “A/E glass” filter (PALL Life Sciences, NY, USA; 142mm diameter) using a peristaltic pump, which prevented microzooplankton and larger particles from passing through with bacteria [35]. We then conducted a full-factorial experiment on each sample which included C, N, P, and salt (S) as factors. Thus, there were in total 16 different treatments (control, +C, +N, +P, +CN, +CP, +NP, +CNP, control+S, +CS, +NS, +PS, +CNS, +CPS, +NPS, +CNPS), and replication was made in duplicates. Sample sub-volumes of 5 mL were added to 5-mL glass vials. After, 25 μL of standard nutrient solutions (concentrations 1000 mg C L^−1^, 100 mg N L^−1^, and 10 L^−1^ 100 mg P L^−1^) were added to the 5-mL vials in order to obtain the desired concentrations. Amendments of C were made by adding C_6_H_12_O_6_ (glucose) to a concentration of 5 mg C L^−1^. Amendments of N were made by adding NH_4_NO_3_ (ammonium nitrate) to a concentration of 0.5 mg N L^−1^ and amendments of P were made by adding Na_2_HPO_4_ (disodium phosphate) to a concentration of 0.05 mg P L^−1^, while control treatments received no amendments. The three elements were added in concentrations sufficiently large to remain in excess throughout 72 h of incubation. We choose a 72-h incubation period since, based on previous studies, we observed that bacteria need approximately 48 h to reach peak BP, and in addition, bacteria need time to adapt to the experimental temperature. Salt was initially considered as a factor, as differences in salinity levels for samples taken during periods of high or low flow could impact microbial substrate uptake [32]. To test whether salt influenced C, N, or P bioavailability, we added an artificial salt mixture (slightly oversaturated stock solution: 3.02 mol NaCl L^−1^, 0.065 mol KCl L^−1^, 0.015 mol Na_2_CO_3_ L^−1^, 0.265 mol CaCl_2_ L^−1^, 0.162 mol MgCl_2_ L^−1^, 0.183 mol MgSO_4_ L^−1^) and raised salinity by 5 units. We tested the effect of salt with a generalized linear mixed model (GLMM; see “Statistical analyses” section for detailed description). Because the effect of salt was not significant for BR for site B3 (GLMM; *F*_1,219_ = 0.000, *p =* 0.983) neither B7 (GLMM; *F*_1,219_ = 1.990, *p =* 0.160), nor for BP for site B3 (GLMM; *F*_1,219_ = 1.612, *p =* 0.206) nor for B7 (GLMM; *F*_1,219_ = 1.802, *p =* 0.181), we removed salt from the statistical analysis and pooled the replicates that did not contain additional salt with those to which salt was added. This resulted in a full-factorial design with three factors and quadruplicate samples (thus eight treatment combinations: control, +C, +N, +P, +CN, +CP, +NP, +CNP).

### Bacterial Respiration and Production Measurements

After preparation, the vials were tightly closed and connected to a dynamic luminescence quenching-based O_2_ sensing system (Sensor Dish Reader, SDR2; PreSens GmbH, Germany) and placed in the dark at 20 °C. The system recorded oxygen concentrations every 5 min for 72 h. Estimates of BR rates were determined for each treatment and date as the average slope of four linear regressions. The respiratory quotient was set to 1, which is within the range of estimates for rivers [36, 37] and estuaries [38, 39].

After terminating the BR measurements, 1.2 mL of each sample vial was pipetted into sterile 1.5-mL Eppendorf tubes which were then used to measure BP with the ^3^H-leucine incorporation method Smith and Azam [40], modified by Karlsson, Jansson, and Jonsson [41]. The ^3^H-leucine was added to a final concentration of 30 nM (specific activity was 120 Ci mmol^−1^, Perkin Elmer). Leucine incorporation was determined by incubation for 1 h in the dark at 20 °C. Incubations were stopped with trichloroacetic acid additions of 5% (*w*/*v*). A bacterial pellet was formed by centrifugation for 10 min at 14,000 rpm and then rinsed with 5% TCA. After addition of 1.2 mL of scintillation cocktail (PerkinElmer), radioactivity was determined on a Wallac WinSpectral 1414 Scintillation counter (PerkinElmer). Incorporation of ^3^H-leucine into protein was calculated using an intracellular dilution factor of 2 [40]. One blank vial (pretreated with TCA before the addition of leucine) was measured per 24 analyzed samples.

### Environmental Data

We retrieved data on physicochemical and biological variables for the two sites from the Marine Environmental Monitoring Program and archived by SMHI. We also compiled data on river discharge from SMHI. Discharge was measured on a daily basis at the Torrböle station which is the closest monitoring station from the river outlet, located 25 km upstream [33].

### Statistical Analyses

We used a GLMM to test for significant differences in BR and BP enriched treatments relative to the controls among the separate treatments, i.e., enrichment combinations (+C, +N, +P, +CN, +CP, +CNP), over time. Time was included in the model as a random variable to account for temporal autocorrelation. The GLMM can be used to analyze nonnormally distributed data and has the potential to account for random effects terms, such as time [42]. The same type of model was also used to test significance of main effects (C, N, and P), two- (C × N, C × P, N × P) and three-way interactions (C × N × P) over time. Analyses were conducted with SPSS® statistical software (v. 22; IBM Corporation, Armonk, NY, USA) with critical *p* value set to 0.05. Additionally, we explored the regulation of BR and BP by performing a principal component analysis (PCA) using XLSTAT Version 2017.5 (AddinSoft, Paris, France). Missing data (12 observations; Table 1) were estimated with nonlinear iterative partial least squares algorithm prior to the PCA [43]. Data were automatically centered and standardized with the PCA. Lastly, we calculated relative increases in marginal means as measures of main effect size for the strongest main effects observed on BP and BR. For example, the main effect of P on BP was determined by dividing the marginal mean of all treatments in which P was added (P, P+N, C+P, C+N+P) by the marginal mean of all treatments in which P was not added (control, C, N, C+N). Main effects (relative increases in marginal means) were then correlated to the principal components (PCs), which are in turn informative of patters of variation of potential explanatory variables.

## Results

Ambient conditions at the Öre estuary varied during the experimental period (Table 1). Concentrations of DOC varied between 343–458 μM, DON 9.9–24.2 μM, and DOP 15.4–24.4 μM. The average ratio of DOC to TN (DOC/TN) was 21 (± 3.8 SD), DOC/TP was 897 (± 375 SD), and TN/TP was 42 (± 10 SD). Salinity was lowest in the beginning of May (0.2) concomitant with the highest river discharge (75.6 m^3^ s^−1^), and highest during winter (5.1) when low riverine inputs occurred (8.4 m^3^ s^−1^).

**Fig. 2:**
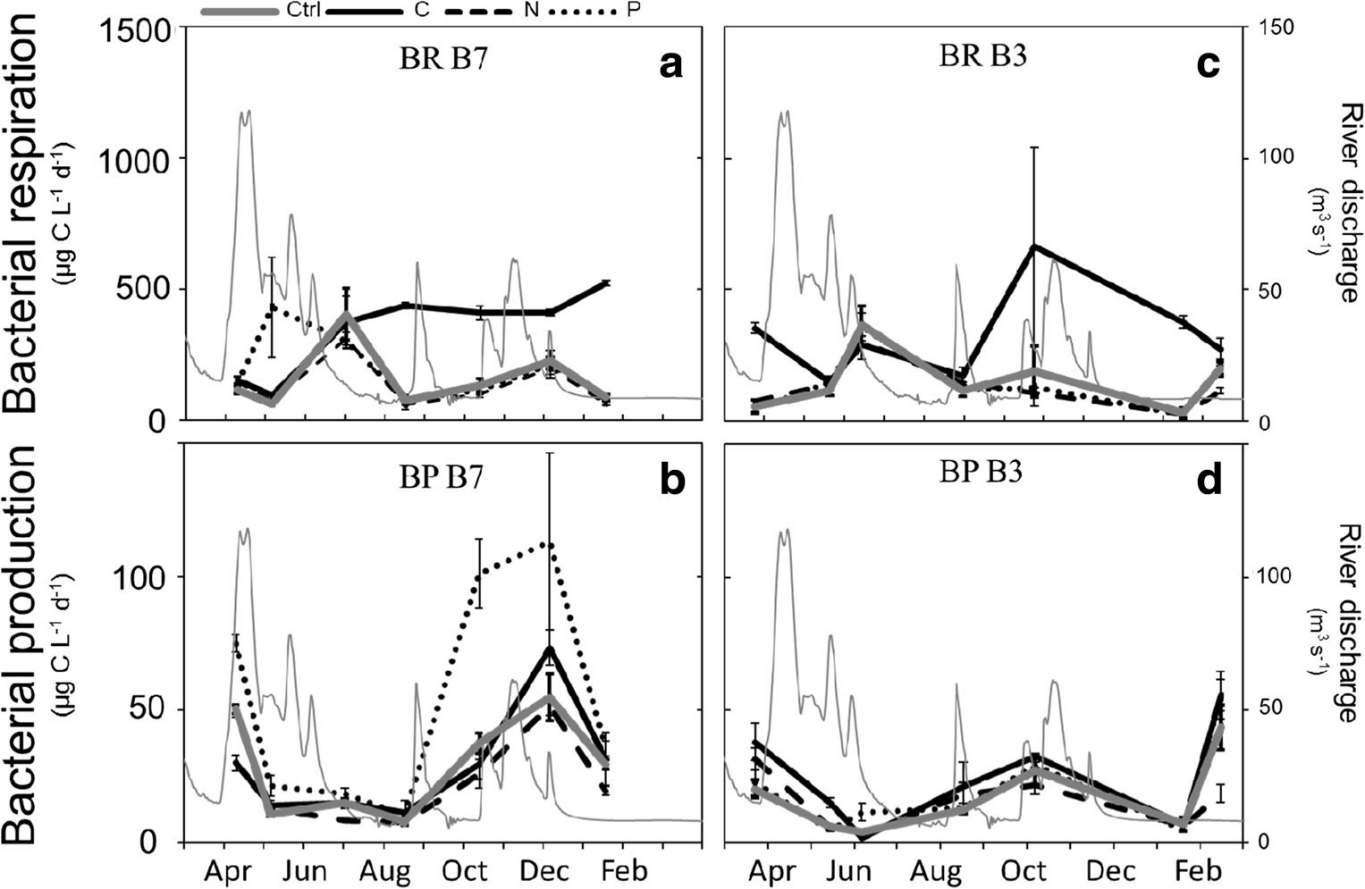
Rates of bacterial respiration (BR) and bacterial production (BP) in incubations with single amendments of carbon (C), nitrogen (N), or phosphorus (P) for B7 and B3 stations of the Öre estuary in the Baltic Sea, Northern Sweden. Ctrl control treatment (no amendments). Data are means of four replicate incubations

Responses of BR and BP to single and combined element additions varied qualitatively between the two study sites. Stimulation of BR in samples from site B7 occurred in response to single C additions (GLMM; *F*_1,54_ = 30.589, *p <* 0.001; Fig. 2a) and thus confirmed our first hypothesis. Rates of BR increased on average 2.2 times in response to C additions and compared with nonenriched controls; however, BR rates did not increase in response to N or P single amendments (GLMM; *F*_1,54_ = 1.647, *p =* 0.205; *F*_1,54_ = 0.970, *p =* 0.329, respectively). At site B3, BR rates were also stimulated by C additions (GLMM; *F*_1,54_ = 0.387, *p <* 0.537; Fig. 2c), while N and P did not have an effect (GLMM; *F*_1,54_ = 0.387, *p =* 0.537, *F*_1,54_ = 0.569, *p =* 0.454, respectively). In regard to BP, at site B7, P amendments alone had a strong stimulating effect, increasing BP by 1.8 times on average (GLMM; *F*_1,54_ = 15.661, *p <* 0.001; Fig. 2b). Single N amendments decreased BP (GLMM; *F*_1,54_ = 5.618, *p <* 0.021), while amendments of C had no significant effect (GLMM; *F*_1,54_ = 0.001, *p =* 0.973). At site B3, single C additions stimulated BP (GLMM; *F*_1,54_ = 7.448, *p <* 0.009; Fig. 2d), whereas single additions of N and P had no effect (GLMM; *F*_1,54_ = 0.854, *p =* 0.360, *F*_1,54_ = 2.346, *p =* 0.131, respectively).

**Fig. 3:**
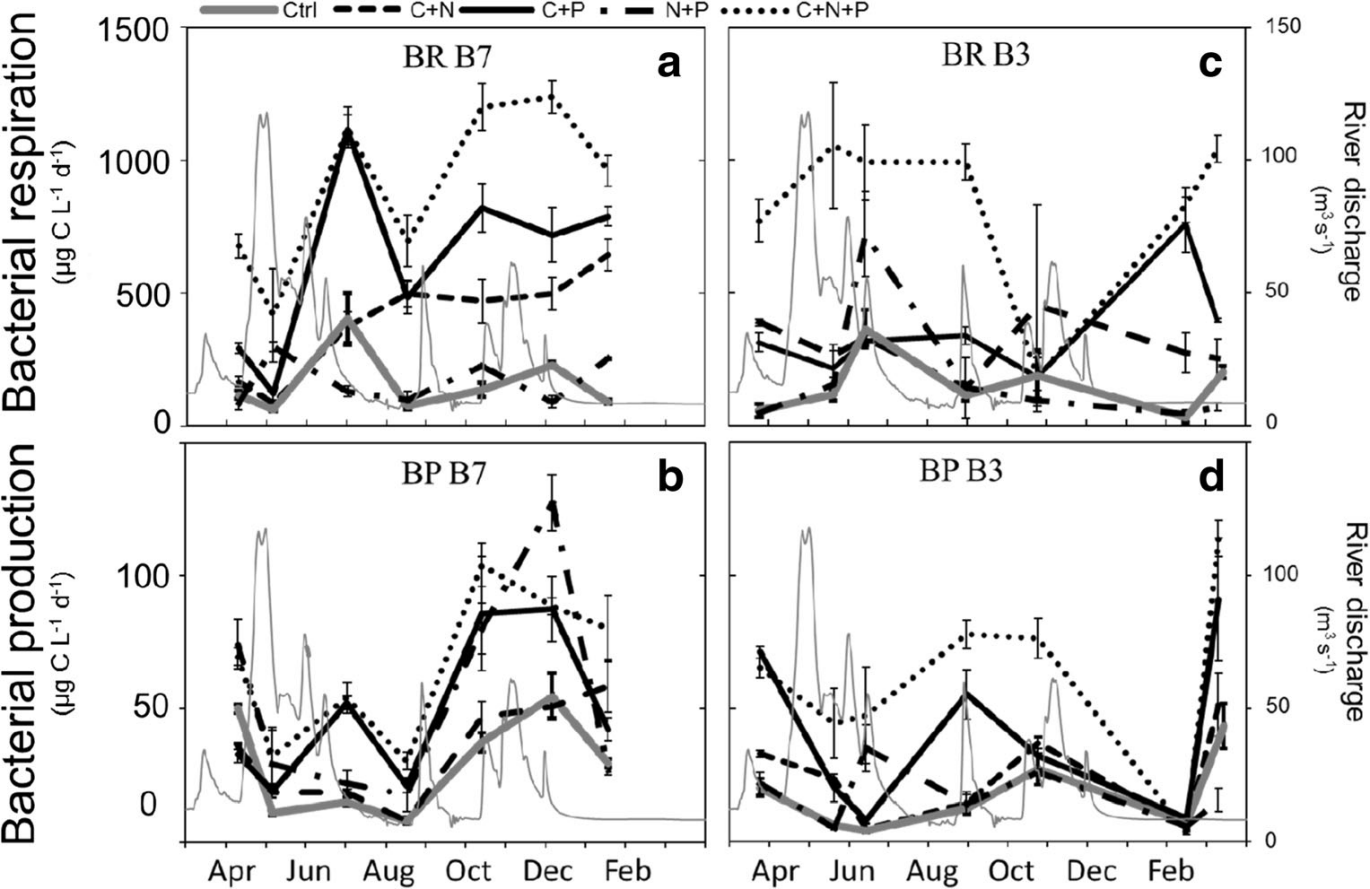
Rates of bacterial respiration (BR) and bacterial production (BP) in treatments amended with nitrogen and phosphorus (N+P), carbon and nitrogen (C+N), carbon and phosphorus (C+P), or C+N+P. Ctrl control treatment (no amendments). Data are means of four replicate incubations

Combined additions of C+N led to an average increase in BR rates of approximately 1.9 times at site B7 (GLMM; *F*_1,54_ = 37.439, *p <* 0.001; Fig. 3a), additions of C+P increased BR rates by 3.9 times (GLMM; *F*_1,54_ = 109.735, *p <* 0.001) and additions of C+N+P increased BR by 5.7 times (GLMM; *F*_1,54_ = 222.754, *p <* 0.001). At site B3, addition of C+N increased BR on average 2.6 times (GLMM; *F*_1,54_ = 14.596, *p <* 0.001; Fig. 3c), addition of C+P increased BR on average 2.4 times (GLMM; *F*_1,54_ = 22.075, *p <* 0.001) and C+N+P additions increased BR by 5.5 times (GLMM; *F*_1,54_ = 118.959, *p <* 0.001). Bacterial production at site B7 increased on average 1.8 times in response to N+P additions (GLMM; *F*_1,54_ = 26.641, *p <* 0.001; Fig. 3b), 1.5 times in response to C+P additions (GLMM; *F*_1,54_ = 16.892, *p <* 0.001), and 2.2 times in response to C+N+P additions (GLMM; *F*_1,54_ = 90.930, *p <* 0.001). At site B3, BP increased on average 1.4 times in response to C+N additions (GLMM; *F*_1,54_ = 7.851, *p <* 0.01; Fig. 3d), 2.4 times in response to C+P additions (GLMM; *F*_1,54_ = 23.788, *p <* 0.001) and 3.6 times in response to C+N+P additions (GLMM; *F*_1,54_ = 80.788, *p <* 0.001).

**Fig. 4:**
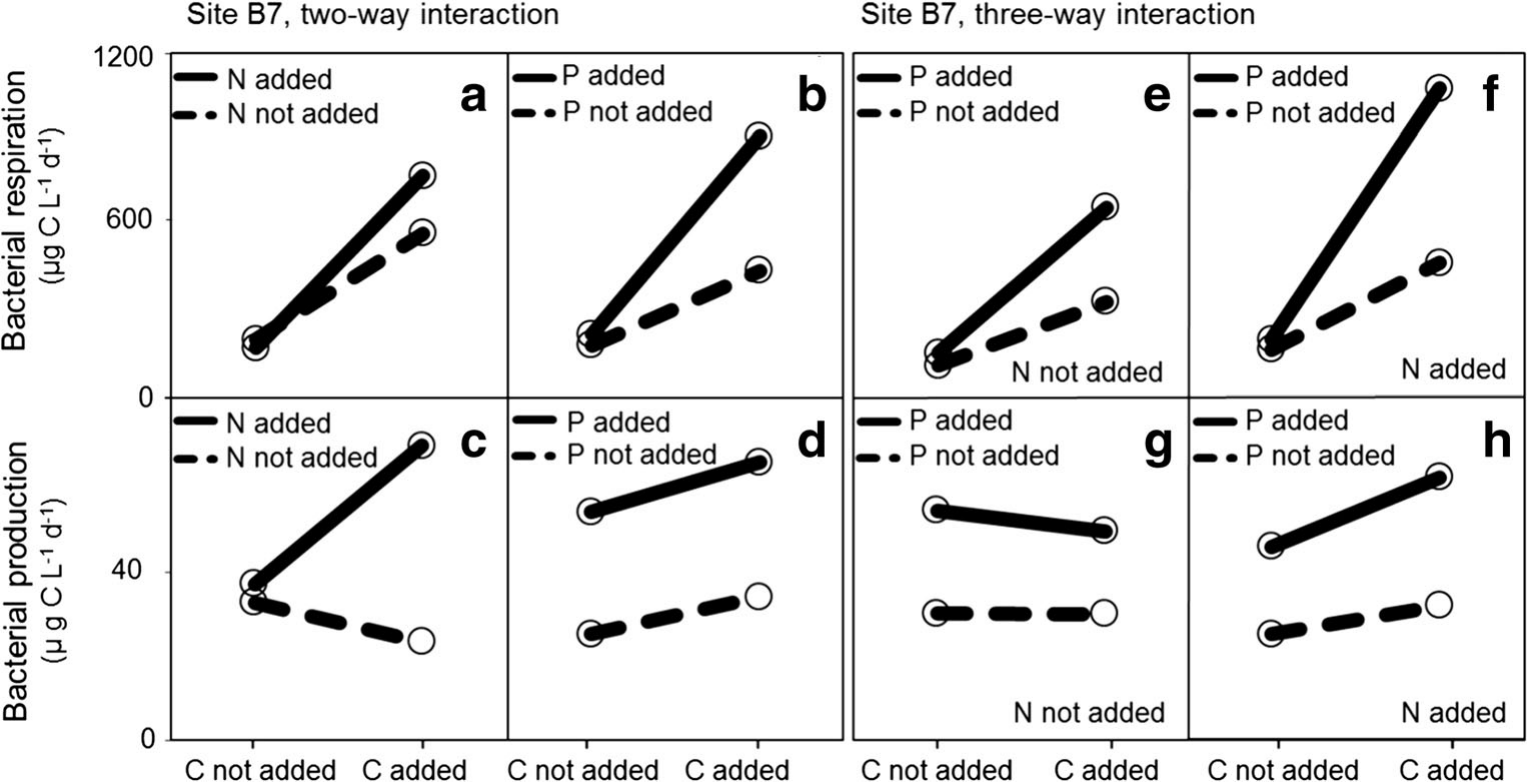
Marginal mean profile plots for bacterial respiration (BR) and bacterial production (BP) for site B7. The marginal mean values when an element is not added are shown as average observed rate in all treatments of the full-factorial experiment where the element was not added, while the response when an element was added represent the average response of all treatments that were amended with the element. Profile plots are shown for carbon (C) and nitrogen (N) interactions (**a**, **c**), C and phosphorus (P) interactions (**b**, **d**), and interactions between C, N, and P (**e**–**h**)

**Fig. 5:**
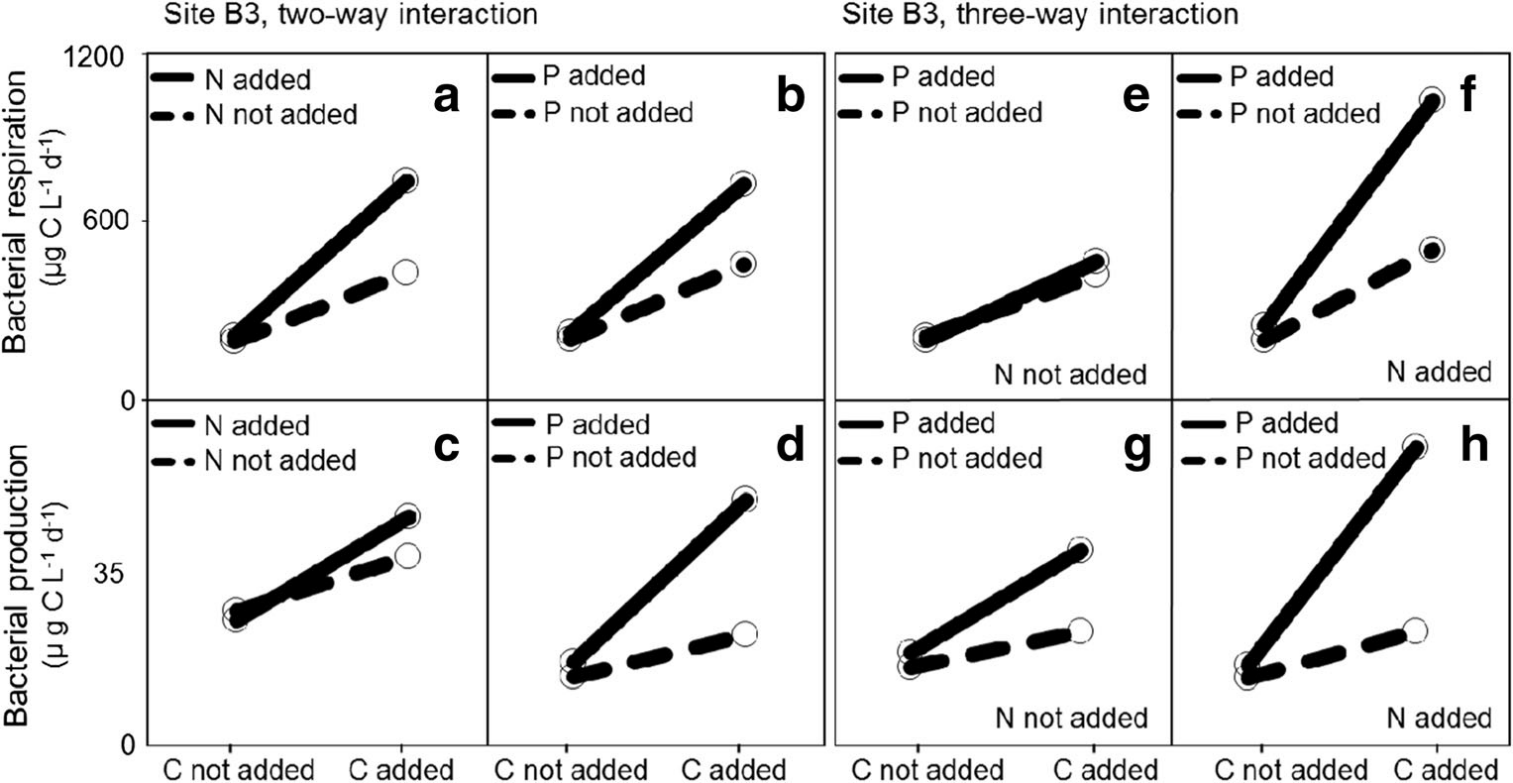
Mean marginal mean showed as profile plots for bacterial production and bacterial respiration for site B3. Profile plots are shown for carbon (C) and nitrogen (N) interactions (**a**, **c**), C and phosphorus (P) interactions (**b**, **d**), and interactions between C, N, and P (**e**–**h**)

We observed a C × N × P interaction on BR at both study sites (Figs. 4f and 5f; Table 2), thus confirming our third hypothesis. Phosphorus was, nonetheless, significant as a main effect on BP at site B7 (Fig. 4d, g, h; Table 2), and a weak two-way interaction between C and N on BP was also observed at the same site (Fig. 4c; Table 2). At site B3, C+N+P showed a positive three-way interaction on BP (Fig. 5h; Table 2).

**Table 2:** Main effects, two- and the three-way interactions from generalized linear mixed model analyses for bacterial production (BP) and bacterial respiration (BR) for B7 and B3 coastal stations in response to additions of carbon (C), nitrogen (N), and phosphorus (P). Interactions were considered significant for *p* < 0.05

Treatments	BPB7		BPB3		BRB7		BRB3	
	*F*_*df* = 1,216_	*p*	*F*_*df* = 1,216_	*p*	*F*_*df* = 1,216_	*p*	*F*_*df* = 1216_	*p*
C	2.106	0.148	83.406	< 0.001	272.368	< 0.001	105.558	< 0.001
N	2.757	0.098	2.902	0.090	8.608	< 0.01	20.035	< 0.001
P	96.324	< 0.001	44.688	< 0.001	74.279	< 0.001	15.728	< 0.001
C × N	5.703	< 0.05	7.869	< 0.05	14.829	< 0.001	15.728	< 0.001
C × P	0.032	0.859	29.326	< 0.001	57.448	< 0.001	11.941	< 0.001
N × P	2.549	0.112	5.108	< 0.05	4.842	< 0.05	12.264	< 0.001
C × N × P	0.522	0.471	5.616	< 0.05	6.281	< 0.05	6.868	< 0.01

**Fig. 6:**
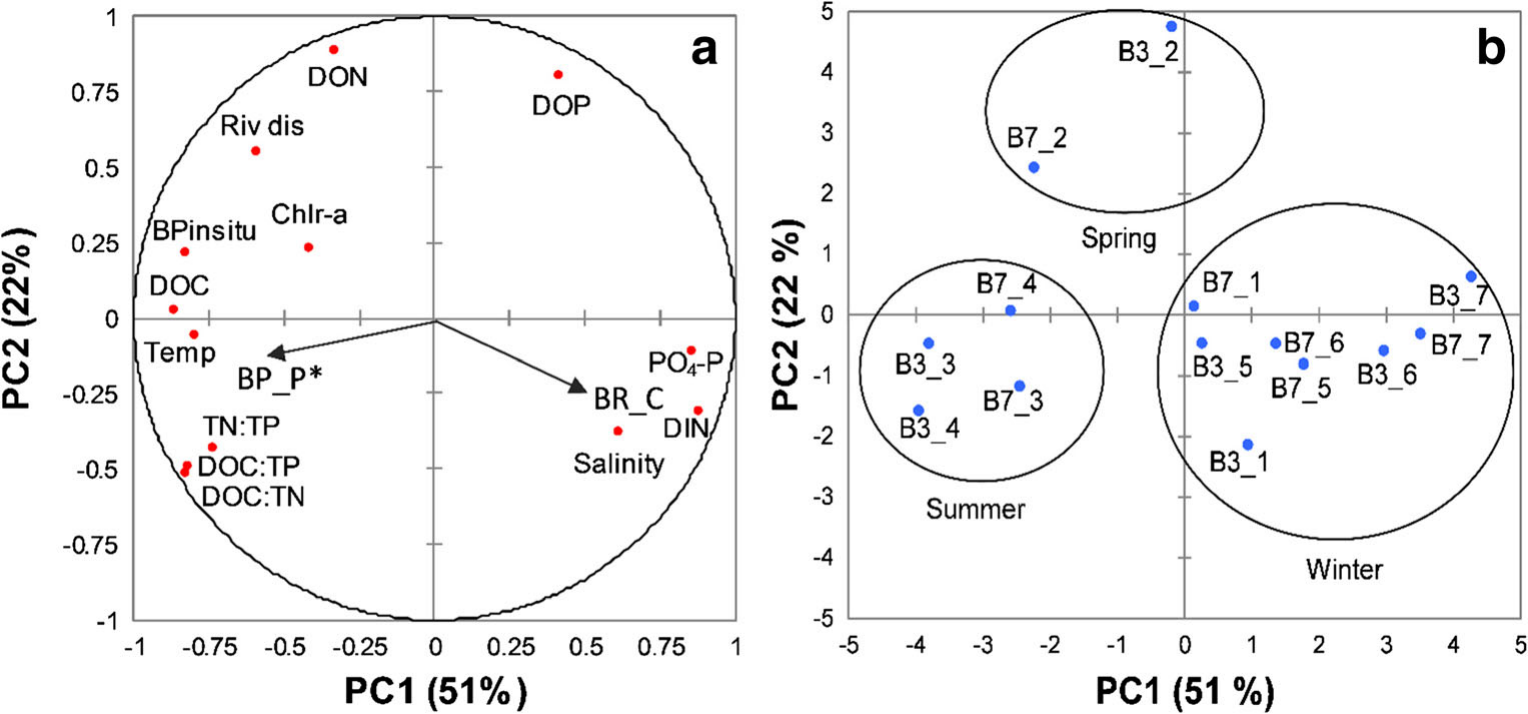
Principal component (PC) loading plot, showing PC1 and PC2 for 13 potential explanatory variables at the two estuarine studied sites (Table 1; abbreviations are defined in Table 1). Vectors show Pearson correlations between PC scores and relative increase in marginal means for bacterial respiration (BR) in response to C enrichment and for bacterial production (BP) in response to P enrichment. The asterisk denotes a significant (*p* < 0.05) correlation between the marginal mean and the horizontal axis. Ellipses show samples grouped by season

The PCA extracted two significant PCs which together explained 73% of the total variance (Fig. 6a). The PC1 (51%) was characterized by high negative loadings for variables related to river discharge (e.g., chlorophyll-a, DOC, in situ BP) and strong positive loadings for variables representing marine-like conditions, such as high inorganic nutrients concentrations and high salinity. In turn, PC2 (22%) was characterized by positive loadings on the part of the axis related to river discharge and organic nutrients, and by negative loadings for the remaining variables (e.g., temperature, element ratios, inorganic nutrients, salinity). Both coastal sites showed a large variation in the 13 variables included in the PCA, which was demonstrated by the scattering of the sites scores within the ordination space of the scores plot (Fig. 6b). In general, samples with positive scores on PC1 were related to low flow conditions, while negative scores on PC1 were indicative of high flow conditions. Samples with negative PC1 and positive PC2 (B7_3, B7_4, B3_3, B3_4; see Table 1 for sample legend) had in general high water temperatures, high resource ratios and high flow which are typical summer conditions. Samples with positive PC1 and negative PC2 (B7_5, B7_6, B3_1, B3_5) were related to pre- and post-wintering conditions such high salinity and high concentrations of inorganic nutrients. However, some winter samples (B7_1, B3_5, B7_5, B7_6) were not well explained by any of the PCs, as shown by the low scores on both axes. We further explored how the 13 estuarine variables relate to marginal means for BR and BP rates in response to additions of C and P, respectively. Relative marginal mean increase for BP upon P addition was negatively correlated with PC1 (Pearson’s correlation, *r* = − 0.557, *n* = 14, *p <* 0.05), thus related to high riverine discharge (Fig. 6a). There was no significant correlation between BP marginal mean increase in response to P and PC2 (Pearson’s correlation, *r* = −0.149, *n* = 14, *p =* 0.611). Marginal mean increase for BR upon C addition was positively related to PC1; however, this relationship was only marginally significant (Pearson’s correlation, *r* = 0.513, *n* = 14, *p =* 0.061). There was no correlation between BR marginal mean increase and PC2 (Pearson’s correlation, *r* = 0.254, *n* = 14, *p =* 0.380).

## Discussion

The predominant qualitative patterns of the single and interactive effects of increasing C, N, and P on bacterioplankton metabolism in the Öre estuary has to our knowledge been determined for the first time in this study. While it is difficult to predict how climate change will affect the riverine export of bioavailable nutrients and organic carbon to estuaries, our results shed light on the role of resource availability on the regulation of bacterial metabolism, which can be used to speculate how estuarine systems will respond to different scenarios of DOM and nutrient increase. Our study shows that P is a key nutrient, controlling BP in the Öre estuary, as BP was found primarily limited by P. Bacterial respiration increased in response to additions of C alone, and particularly in response to the addition of C combined with P and/or N. Because increases in terrestrial C and P fluxes are expected in the area, an increase of BP is expected as well as an increase in BR of terrestrially derived C.

### Single Addition Effects

Additions of C alone had a large positive effect on BR rates at both study sites, while additions of N and P alone had no effect. These findings agree with our first hypothesis by confirming the role of C as primary limiting element for BR [3], which is consistent with several other studies showing that inorganic nutrients alone tend to have a marginal impact on BR in estuaries [44–46]. Interestingly, bacteria in our study were able to respire the added C, even when bacterial growth was strongly limited by P (Fig. 2a, b). Similar findings have been reported in a previous study where the relative allocation of assimilated C to respiration was shown to be very high for P starved bacteria [47]. During periods of P limitation, the uptake of C is mostly directed to respiration in order to energize processes needed to maintain cellular integrity, such as osmotic regulation, renewal of macromolecules, and membrane transport functions [3, 48, 49].

We found that BP was stimulated by single P additions at site B7. This contrasts our second hypothesis that additions of P and C in combination are required to enhance BP at the Öre estuary, yet agrees with one previous report of P limitation of bacterial productivity in the Öre estuary [24]. Empirically, BP has been shown to increase with P concentrations in a wide range of low- and high-productive surface waters [50, 51], but results from correlational studies are not consistent [52]. Often, the identification of P as main regulator of BP is hindered by the fact that the export of terrestrially derived DOC co-occurs with that of inorganic and organic P [9, 53], both of which are known to be highly bioavailable at Baltic Sea river mouths [54]. A large number of studies have addressed the direct effects of increasing DOC on aquatic ecosystems, but increased P fluxes driven by the increase in riverine DOM exports are often neglected [55]. While our study shows that BP at the Öre estuary is P limited, it is possible that the organic P content of the riverine DOM alleviates P limitation of BP [Fig. 6a; 5]. Thus, riverine DOM inputs can relieve limitation on BP, however not by delivering C as previously suggested, but rather by providing some bioavailable P to the estuary. In the Baltic Sea, empirical studies on nutrient limitation suggest that most basins are N-limited, although in coastal areas highly influenced by riverine freshwater of TN/TP ratios well above Redfield’s, P limitation tends to occur [56].There was a shift in BP limitation from site B7 to site B3. At site B3, BP was no longer primarily limited by P but instead there was a small yet significant response to C amendments. The higher average salinity levels of site B7 in relation to site B3 (Table 1), combined with a closer distance between site B7 and the river mouth (Fig. 1), suggest that freshwater inflows reach site B7 faster than site B3. Thus, a change in BP limitation could indicate that bioavailable riverine C was utilized at site B7, but consumed before reaching B3. The uptake of C for BR and BP may have resulted in an overall greater loss of bioavailable C, in relation to P, thus leading to C-limited BP at B3. Photochemical processing likely contributed to further carbon losses and enhanced microbial degradation during transit of DOM through the estuary [13]. Another possibility is that C could have been removed from the water column due to aggregation and subsequent sedimentation; however, C sedimentation at the Öre estuary has been shown low [31]. The fact that BP rates were on average higher at site B7 (Fig. 2b) than at site B3 (Fig. 2d) also suggests that riverine DOM first reached site B7 and became C depleted, likely due to microbial decomposition, during its transit to site B3.

### Combined Additions and Element Interactions

Combined additions of C and nutrients (C+N, C+P, C+N+P) led to large increases in BR rates at both sites, where significant C × N × P interactions were also determined. These results confirm our third hypothesis of costimulation between the three elements. The fact that N and P (together with C) costimulated BR is not surprising, since N and P are critical nutrients to several structural and metabolic cell functions, such as energy transport and synthesis of proteins [3]. In line with previous work [44, 57, 58], our study suggests that higher BR rates can be expected if increases in C and nutrients occur simultaneously.

Combined element additions (C+N, C+P, N+P, C+N+P) led in general to increased rates of BP at both sites. While P was significant as main effect on BP at site B7 (no interaction), at site B3, we observed a positive three-way interaction between C, N, and P. The fact that P had a unique effect on site B7 indicates that riverine inflows led to a strong P-limitation at the site. Despite C sedimentation within the estuary being low [31], a large share of the P transported in the river runoff may have been removed from the water column and became unavailable to the estuarine microbial pool due to formation of iron-phosphate complexes, and subsequent sedimentation [31]. Microbial utilization combined with physical and chemical immobilization of riverine substrates within the estuary may explain the observed filtering effect, which resulted in relatively low supply of bioavailable organic carbon at site B3, where C, N, and P interactively limited BP. Due to the different patterns of limitation observed, our study suggests that the effect of riverine DOM inputs on BP was different within the estuary. Our findings can, nonetheless, be extrapolated to larger scales and to systems that heavily influenced by external inputs of organic matter and expected to (1) experience increases in terrestrial dissolved organic matter concentrations and (2) have a similar climate, landscape, and hydrographic characteristics [59].

### Seasonal and Spatial Patterns, Environmental Controls

Patterns in resource limitation of bacterioplankton metabolism were further clarified in the PCA which made possible to visualize how our 14 samples related to the 13 variables representative of environmental conditions at the estuary (Table 1; Fig. 6b). The positive correlation between the main effect of P on BP (relative marginal mean response to P additions) and PC1, which is in turn related with discharge conditions, further supports that riverine inflows induce P limitation of BP in the estuary (Fig. 6a). In periods of high discharge such as during spring and early summer (Table 1; Fig. 6b) P limiting conditions were observed (Fig. 2b), even though P limitation was to some extent relieved during the same period (Figs. 2b and 6a, b). Thus, freshwater riverine inputs are both the main source of P and the cause of P limitation in the estuary.

The main effect of C on BR (relative marginal mean response to C additions) was negatively related to PC1, although this relationship being only marginally significant. During periods of low riverine discharge such as during winter (Table 1, Fig. 6b), C limitation of BR was greater, likely due to the low input of organic C, which resulted in a greater effect of C additions on respiration rates during this period. Together, our experimental results in combination with PCA suggest a strong terrestrial riverine inflow influence on microbial metabolic processes and that discharge is a strong regulator of the intra-annual variability conditions at the Öre estuary.

Resource stoichiometry indicators such as total C/N/P ratios were high (average of 896:21:1; molar) in comparison to the C/N/P of natural aquatic and cultured bacteria [50:10:1; 50, 60, 61], which may suggest P-limitation of BP. In the Öre estuary, it has been reported that during periods of high precipitation, severalfold increases of C and N loading occur, while increases of P are moderate [9]. Others have also reported relatively higher increases of C and N fluxes in comparison to those of P, which may in the future result in global patterns of P-limitation of marine ecosystems [62, 63]. Nonetheless, even if small, increases in P loadings can enhance BP, particularly because total P has been shown roughly 25 times more available than DOC [54]. Consequently, DOC/TN and DOC/TP ratios are much higher than correspondent bioavailable C/N and C/P ratios [54]. We expect a stimulation of BP in the future to the extent that some bioavailable P will increase along with increasing loadings of terrestrial DOC.

### Methodological Considerations

In this study, we added specific compounds not to mimic the natural DOM, but rather to relieve resource limitation such that the maximum potential resource limitation could be determined. Thus, rates of BP and BR obtained are specific to the compounds utilized here and not representative of the whole range of compounds that can typically be found in the DOM. In addition, BR rates were obtained during a 72-h incubation performed at the 20 °C. It must be thus acknowledged that such rates cannot be directly transferred to natural boreal systems. The BP rates were however less influenced by the 20 °C incubation and compare to rates reported in other Baltic Sea nutrient enrichment experiments performed in situ [22, 64]. The effect of temperature on BR and on BP rates found here is in line with previous findings, which suggest that temperature is the main regulator of BR but not of BP [26, 65].

After 72 h, it is possible that the composition of the bacterial community at the end of the experiment shifted from the initial community composition. In this study, we did not have the opportunity to determine potential changes in the bacterial community composition; however, we do not think that these changes impacted our results since our BP estimates are identical to BP estimates from in situ studies [22, 64]. We did observe a boost on BR rates during incubation, however such increase was expected since the temperature used in our experiment was higher than in situ temperatures. Yet changes in bacterial physiology may occur without changes in the bacterial community composition [66].

### Results in an Environmental Change Context

Global changes are anticipated for the next century, with varying nature and magnitude across different geographical regions [67]. Together, changes in climate, land use, and biogeochemistry will result in changes in the terrestrial runoff which in turn will impact the equilibrium of key processes such as production and respiration. Climate scenarios for northern Sweden forecast an increase in precipitation all year around (http://www.smhi.se/klimatdata/klimatscenarier/Framtidens-klimat); thus, a uniform increase in terrestrial riverine runoff throughout the year should be expected [11]. In this study, we show that if predicted increases of DOM loading bring an increase of estuarine P influx, BP in the coastal zone will likely increase, while an increase in BR may occur in response to increasing C loadings. Based on Wikner and Andersson's [9] study conducted at the Öre estuary during extremely wet years, primary productivity decreases, while higher loadings of C, N and to a smaller extent P were observed. These findings combined with the ones from our study thus suggest that BP will increase and that BR of terrestrially derived C will in the future also increase.

Increases in BR imply a larger DOM-induced dissolved oxygen removal oxygen removal from the Baltic Sea waters, which currently amounts to more than one million tons per year [68]. Thus, the current hypoxic conditions in the northern part of the Baltic Sea may increase, particularly in coastal waters where the biological activity is large (hotspot) and where water column stratification is strong [69]. This is alarming since the Baltic Sea is at present the world’s widest marine area undergoing eutrophication-driven hypoxia [70]. Coupled to a depletion of O_2_, an increase of CO_2_ emissions and of CO_2_ concentrations in the surface waters of the Baltic Sea should be expected. The increase of terrestrial DOM is expected to mainly favor the planktonic heterotrophs. Thus, an increased C metabolism through the microbial loop may possibly decrease the amount of energy transferred to higher levels of the food web [71].

In summary, our study evidence the single role of C for BR limitation and the role of P for BP limitation. Thus, to the extent that terrestrial DOM brings an increase in bioavailable C, BR of terrestrially derived C will increase. In contrast, an increase of BP will require freshwater delivery of bioavailable P. Our results are in line with the increasingly recognized role of terrestrial DOM exports for the regulation of estuarine microbial heterotrophic metabolism, and critical for the prediction of the effects of climate change and potential feedback mechanisms at the coastal zone.
